# Determinants of preventive sexual behaviours among first year university students in Beira city, central Mozambique: a cross-sectional study

**DOI:** 10.1186/s12978-023-01733-6

**Published:** 2024-01-08

**Authors:** Arlinda Basílio Zango, Sarah E. Stutterheim, Nanne de Vries, Rik Crutzen

**Affiliations:** 1 Departamento de Investigação, Faculdade de Ciências de Saúde, Universidade Católica de Moçambique, Beira, Mozambique; 2 Department of Health Promotion, Faculty of Health, Medicine and Life Sciences, Care and Public Health Research Institute, Maastricht University, Maastricht, Netherlands

**Keywords:** Sexual health, Sexually transmitted infections, HIV, Young adults, Mozambique, Saúde sexual, Infecções transmitidas sexualmente, HIV, Jovens adultos, Moçambique

## Abstract

**Background:**

Understanding determinants of preventive sexual behaviours is important for intervention efforts to support these behaviours and, thereby, reduce STIs and HIV burden. In general, there is limited insight into determinants of preventive behaviours among university students in Mozambique. Therefore, this study set out to assess both the prevalence and the determinants of condom use and voluntary counselling and testing (VCT) service use in first year university students.

**Methods:**

We conducted a cross-sectional study in May–September 2021, at the *Universidade Católica de Moçambique* and the *Universidade Licungo*, in Beira central Mozambique. We collected data on sociodemographic characteristics, heterosexual relationship experiences and personal determinants posited to be associated with condom use and VCT service use. We included 819 participants, who were selected using a clustered and random sampling design. We used Pearson's chi-square test to compare proportion and estimate the crude odd ratio as the effect size measure at 95% confidence interval, and Confidence Interval-Based Estimation of Relevance to determine correlation coefficients of means and the behaviours of interest at 95% confidence interval.

**Results:**

Condoms were used by 96.1% of male participants and 95.0% of female participants. Additionally, 55.1% of male participants and 57.5% of female participants had previously used VCT services. Condom use was associated with discussing sexuality with mother, and self-efficacy for condom use negotiation, and negatively associated with attitudes that condoms reduce pleasure. VCT service use was associated with discussing sexuality with mother, sexual debut, having a sexual partner, and being in what they consider an important heterosexual relationship. Knowledge, attitude, self-efficacy and subjective norms were weakly associated with VCT service use.

**Conclusion:**

In first year university students in Mozambique, reported condom use was high but VCT services were only used by about half of the participants. Interventions aiming to increase VCT service use should focus on improving communication between parents and their adolescent or young adult children, providing personalized risk information, demonstrating that VCT service use is pleasant and non-judgmental, improving users’ confidence to schedule a visit, and preparing users for possible positive testing results.

## Portuguese Abstract

**Introdução:**

Compreender os determinantes de comportamentos sexuais preventivos é importante para guiar o desenvolvimento de intervenções que reforçam e apoiam estes comportamentos e, assim, reduzir a carga das infecções transmitida sexualmente (ITSs) e do Vírus de Imunodeficiência Humana (HIV). Em geral, informação sobre os determinantes de comportamentos preventivos entre os estudantes universitários é limitada em Moçambique. Portanto, este estudo teve como objetivo avaliar a prevalência e os determinantes do uso de preservativos e uso dos serviços de aconselhamento e testagem em saúde (ATS) em estudantes universitários do primeiro ano.

**Métodos:**

Realizamos um estudo transversal em Maio—Setembro de 2021, na Universidade Católica de Moçambique e na Universidade Licungo, na Beira centro de Moçambique. Coletamos dados sobre características sociodemográficas, experiências de relacionamento heterossexual e determinantes pessoais considerados associados ao uso de preservativo e ao uso dos serviços de ATS. Foram incluídos 819 participantes, estes foram selecionados usando o desenho amostral de conglomerado e aleatório. Utilizamos o teste qui-quadrado para comparar as proporções, usamos odd ratio para estimativa de tamanho do efeito a 95% de intervalo de confiança. e para identificar os determinantes relevantes usamos a abordagem da estimativa de relevância baseada no intervalos de confiança (CIBER).

**Resultados:**

O uso do preservativo foi declarado por cerca de 96,1% dos participantes homens e 95,0% de participantes mulheres. Mas apenas 55,1% dos participantes homens e 57,5% das participantes mulheres referiram terem usado o serviço de ATS. O uso do preservativo foi associado à discussão da sexualidade com a mãe e positivamente associado à autoconfiança para negociação do uso do preservativo, e negativamente associado à atitude de que, o preservativo reduz o prazer. O uso dos serviços de ATS foi associado à discussão da sexualidade com a mãe, início da vida sexual, ter um parceiro sexual e estar num relacionamento heterossexual que consideram importante. Conhecimento, atitude, autoconfiança e normas subjetivas mostraram-se fracamente associados ao uso de serviços de ATS.

**Conclusão:**

Em estudantes universitários do primeiro ano em Moçambique, o relatado de uso do preservativo foi elevado, mas os serviços de ATS foram usados por apenas cerca de metade dos participantes. Intervenções que visam aumentar a visita e utilização dos serviços ATS devem centrar-se na melhoria da comunicação entre os pais e os seus filhos adolescentes ou jovens adultos, fornecendo informações personalizadas sobre riscos, demonstrando que a utilização dos serviços de ATS é agradável e sem julgamentos, incentivar a autoconfiança dos utentes para agendar uma visita à ATS e preparar usuários para possíveis resultados de testes positivos.

## Introduction

Unhealthy sexual behaviour leads to high prevalence and incidence of sexual transmitted infections (STIs) and HIV among adolescents and young adults (AYA). Despite efforts devoted to preventive action, STIs continue to increase globally (1), from 357 million new cases of the main four curable STIs—gonorrhoea, chlamydia, trichomoniasis and syphilis (2)—in 2012 to 374 million new cases in 2021 (3).

AYA in Sub-Saharan Africa are disproportionally affected by STIs. For instance, among youths aged 15–24 across countries in the Sub-Saharan region, the estimated prevalence ranges were 6.4–11.6% for gonorrhoea, 9–17.8% for chlamydia, 10.5–20.6% for trichomoniasis, and 2.2–10.3% for syphilis (4). In Mozambique specifically, 46% of women aged 18–34 years were diagnosed with at least one STI (5).

AYA are also impacted by HIV. They account for about 1.100 of the 4000 daily new HIV infections (6). In Sub-Saharan Africa, where 51% of new infections were reported in 2021, adolescent girls and young women are specifically at high risk. Additionally, in sub-Saharan Africa, HIV incidence is estimated to be 218 (95% CI 196.4–239.1) cases per 100,000 people, with a range from 2.8 (2.1–3.8) per 100,000 people in Mauritania to 1585.9 (1369.4–1824.8) per 100,000 people in Lesotho (7). In Mozambique, national surveillance data from 2015 revealed an HIV prevalence of 16.6% among AYA aged 15–24 years (8). In Sofala province, where Beira city is located, HIV prevalence among people aged ≥ 15 years old was 13.2% in 2021 (9).

University students are traditionally young adults within the age range of 16–25 years, who face unique challenges as they learn to adapt to changes in academic workload, support networks and new environments. Furthermore, they navigate newly found responsibilities in the context of greater freedom and control over their lives (10, 11). These changes happen concurrently, affecting their bodies and minds as well as their social relationships, and together they can lead to unhealthy or risky lifestyle behaviours, especially in terms of sexual behaviour (12, 13).

Previous studies reporting on sexual behaviour among university students have reported various risk behaviours including unprotected sexual intercourse, multiple sexual partners, alcohol consumption, and early sex debut (14–17). These behaviours have been found to be related to financial needs, lack of sexual education, peer pressure, low risk perception or vulnerability, and impulsivity (11–14, 18, 19).

Insufficient use of STI and HIV counselling, and testing (VCT) services is also considered a sexual risk behaviour among AYA (6). In fact, in Sub-Saharan Africa, only one in five infected adolescent girls (15–24) know their HIV status (20). Some determinants of not using VCT services include not being sexually active, low risk perception, a lack of knowledge on where to seek services, and low comprehensive knowledge of HIV (15, 18).

Additional impacts on sexual behaviours are taboos, poor parent–child communication, parental influences, and peer norms (21–23), as well as economic factors (24, 25). Clearly, sexual behaviour has multiple determinants.

Understanding the relevance of determinants of preventive sexual behaviour is important for intervention efforts. It enables programme designers to develop interventions that leverage evidence-based methods to target relevant, specific and changeable aspects of a determinant, that in turn, influences a behaviour (26–28).

There is currently only limited data available regarding preventive sexual behaviour among university students in Mozambique and limited insight into the determinants of primary (condom use) and secondary (VCT services use) preventive behaviours in this setting. Therefore, this study set out to assess the prevalence, and determinants, of condom use and VCT service use in first year university students in Beira city, central Mozambique.

## Methods

### Setting and study design

This cross-sectional study took place between May and September 2021, in Beira city, Sofala province (in the central region of Mozambique) at two universities: Universidade Católica de Moçambique (UCM) and Universidade Licungo (UniLicungo). We collected data on sociodemographic characteristics, current or recent heterosexual relationship experience, and personal determinants known to be associated with preventive sexual health behaviour, including condom use and VCT service use, namely knowledge, attitudes, self-efficacy, skills, and subjective norms (13, 29, 30).

The personal determinants, and the items assessing them, were selected using the Core Process (31), which comprises both brainstorming and exploring findings from previous research on this topic. The survey was developed in English, translated to Portuguese, the official language of Mozambique, and pre-tested among 26 students. Based on pre-test information, we made adjustment to optimize the language and understanding by future study participants. The survey is available at https://www.frontiersin.org/articles/10.3389/frph.2021.745309/full#supplementary-material data sheet 3. Here, we report on the first wave of data from a larger project (32).

### Participants and sample size

The study population comprised 1938 first year students registered in 2021 academic year. The sample size was 824 students, in order to be able to accurately estimate a correlation coefficient (r) of 0.15, with a 95% confidence interval half-width (w) of 0.10 and assuming a 10% non-response rate (33). In order to identify participants, we used clustered and random sampling in which we selected a total of 23 classes (seven at UCM and 15 at UniLicungo), from which participants were enrolled consecutively, starting from the first class randomly selected, and ending when the target sample size was reached. Inclusion criteria were (1) being a first-year and unmarried university student, studying at one of the two universities in faculties located in Beira city; (2) being aged 16–25 years and (3) having fixed residency in Beira city.

Of the 1938 first year students enrolled in their first year of university, 1318 (68.0%) provided informed consent and 819 (62.1%) of those completed the survey and were included to data analyses. Of the 499 not included, 314 (62.9%) were not eligible, 70 (14.0%) were absent when data were collected, 110 (22.0%) were unavailable because face-to-face education activities were restricted during the COVID-19 pandemic, and five (1.0%) chose not to complete the survey.

### Variables

#### Sociodemographic and living characteristics

We measured sex, age, religion type and frequency of attendance to religious activities. We also measured co-residence with parents using two series of questions (for mother and father) as the following: Is your mother/father alive? If yes, does she/he live in the same household as you? Further, we measured difficulty discussing important issues with parents using a five-point Likert scale: Do you find it difficult or easy to talk with your mother/father about things important to you? (1 very difficult…5 very easy). We then measured frequency of talking with mother or father on a five-point Likert scale (1 never …0.5 a great deal). Lastly, we measured whether AYA discuss sexuality-related matters with their mother or father, and if so, on a five-point Likert scale (1 never…0.5 a great deal) we measured how often.

#### Current heterosexual relationship experience

We used Yes/No questions to check if a participant had a boy/girlfriend and if they had engaged in sexual intercourse. Further, we measured the age at first sexual intercourse, number of boy/girlfriends, and type of relationship (occasional, serious, important and engaged to marriage). Lastly, if applicable, we asked participants, how they would describe the last time they had sex (forced or both willing) and if they had concerns about acquiring STI/HIV infection (Very concerned, somewhat concerned and not concerned).

We asked participants who declared to still be a virgin to indicate the reasons from a list of five options: (a) I do not feel ready to have sex; (b) I have not had the opportunity; (c) I think sex before marriage is wrong; (d) I am afraid of getting pregnant; and (e) I am afraid of getting STI/STD or HIV/AIDS. We also asked these participants to select from a list of four options, their plan to sexual debut: (a) I plan to wait until marriage; (b) I plan until I am engaged to be married; (c) I plan to wait until I find someone I love; and (d) I plan to have sexual intercourse when an opportunity comes along.

#### Personal determinants of condom use

We measured knowledge using six questions: first, we asked participants to select from a list of six items, the consequences of not using a condom; second, we asked them to select from a list of six items, where is the correct place to buy or obtain condom; third, we asked them to select from a list of seven items, what they considered to be a private and effective place to keep condoms; fourth, we asked them to select from a list of five items, which statements represent steps of negotiation for condom use; fifth, we asked them to select from a list of four items, what corresponds to active listening to a partner; lastly, we asked participants to select from a list of seven items, the statements that correspond to the aspects of correct use of condoms.

We measured attitude using 11 questions with seven-point Likert scales, ranging from 1 = disagree completely to 7 = agree completely (e,g. Using a condom during sexual intercourse reduces pleasure…). Self-efficacy was assessed by 10 questions using seven-point Likert scales ranging from 1 = extremely unlikely to 7 = extremely likely (e.g. I am confident to make the decision to use condoms…). Finally, subjective norms were measured with eight questions using seven-point Likert scales ranging from 1 = disagree completely to 7 = agree completely (e.g. Most sexually active students think that it is important to use condoms…).

#### Personal determinants of VCT services use

We used four questions to measure knowledge on VCT services: first, we asked participants to select from a list of five items, the consequences of not using VCT services; second, we asked them to rank their score of agreement that using VCT services reduces spreading of STI including HIV on a five-point scale (1 strongly disagree… 5 strongly agree); third, we asked them to indicate the extent to which they are aware about the risk for STI/HIV infection, using a five-point scale (1 strongly unaware… 5 strongly aware); finally, we asked participants to rate their score of agreement, that they should get help from a health advisor soon for STI/HIV concerns (1 strongly disagree… 5 strongly agree).

We also measured attitude toward VCT service use (e.g. Using STI/HIV screening services is pleasant…), self-efficacy (e.g. I am confident to tell my concerns about STI/HIV to the health provider…) and subjective norms (e.g. Other students like me usually go to the health provider for STI/HIV complaints…) using respectively eight, nine and four items with a seven-point Likert scale as presented above.

### Outcome variables

Condom use: Participants who reported being sexually active were asked if they had used a condom at their last sexual intercourse with two consecutive questions: (a) Were you able to do anything to reduce the risk of acquiring any infection at your last sexual intercourse? (“yes” or “no”) and (b) What did you do? (“Use a condom” and “Take medicine”). Participants that selected “Use a condom” in the second question were labelled as having used a condom at last sexual intercourse while participants that selected “no” in the first question and “take medicine” in the second question were labelled as not having used a condom at last intercourse.

VCT service use: We asked participants if they had ever used VCT services (answer options: “yes”, “no” and “do not remember”). We also asked them to indicate when the last time was they visited a VCT service (“1–4 weeks ago”; “1–3 months ago”; “4–6 months ago”; “A year ago” and “do not remember”). We then converted answers into a binary variable, whereby all participants who selected “yes” to the first question were labelled as having previously used VCT services and who answered “no” or “do not remember” were considered to have not previously used VCT services.

### Procedures

Before data collection, three data collectors were trained for 3 h per day on 3 days. The training explained the study aims and focused on issues related to, for example, how to obtain informed consent, how to approach the participants and how to distribute and collect the surveys. Subsequently, data collection commenced in classrooms during lessons in two sessions occurring on consecutive days. The first focused on the purpose and procedure, as well as informed consent, and the second was to complete the survey. This strategy made it possible for young students (≤ 17 years old) to obtain informed consent from their parents or representatives and older students (18 years or older) had time to understand and make an independent decision to consent.

To minimize missing data and other sources of bias during completion of the surveys, participants were not allowed to chat or to look on the classmate answers thus, in case of doubt about the meaning of specific questions, participants were advised to ask the data collectors for clarification. Data collectors reviewed the completeness of each survey with the participant before they left the classroom. Surveys were also verified by a quality control study member before submission to the data entry unit.

### Statistical analyses

We used Pearson's chi-square test to estimate the relationship between the proportions of condom use, VCT service use and sociodemographic, living characteristics and current or most recent heterosexual relationship experience. The effect sizes were estimated using crude Odd Ratio (OR) and coefficient of correlation (*r*) for categorical and continuous variables respectively, assuming a 95% of confidence interval and p < 0.05 for interpretation of the results. The analyses were performed using IBM Statistical Package for Social Sciences (SPSS) version 27.0.1.

The CIBER approach was used to identify relevant determinants concerning condom use and VCT service use. This approach is based on visualization of confidence intervals for the means and correlation coefficients for all determinants simultaneously (27, 34, 35). It is important to combine univariate distributions of determinants (e.g., means) and their association with the outcome of interest (e.g., correlation coefficients) when selecting relevant determinants. Univariate distributions show the room for improvement regarding each determinant (i.e., how participants score on the scale). This needs to be combined with the association to behavioural outcomes, as those determinants that are associated with behaviour and where there is room for improvement are the most relevant candidate variables to intervene upon. The ‘behaviorchange’ function in R software (36) was used to identify the relevant personal determinants of condom use and VCT service use.

## Results

### Participant characteristics and their relation to condom use and VCT service use

Table 1 presents sociodemographic variables, living characteristics, and current heterosexual relationship experiences, as well as their relation with condom use and VCT service use. Of the 819 participants, 346 (42.2%) were men. Mean age was 19.64 (SD = 2.06) among male participants and 18.59 (SD = 1.65) among female participants. Regarding religion, 338 (41.7%) were Catholic, 56 (6.9%) were Anglican, 47 (5.8%) were Methodist, 282 (34.4%) were followers of other religions, and 67 (8.3%) did not report their religion. Of those who indicated their religion, 265 (35.9%) reported frequently attending religious activities.

**Table 1:** Participant’s characteristics and the relation with condom use and use of voluntary counselling and testing services

Characteristics	N	n (%)	Condom use OR (95% CI)	VCT service use OR (95% CI)
Gender	819			
Men		346 (42.2)	0.76 (0.31–1.87)	1.10 (0.81–1.49)
Women		473 (57.8)		
Age	808		0.06 (−0.38 to 0.50)^*^	0.45 (0.29 to 0.60)^*^
Men: mean (SD)		19.64 (2.06)		
Women: mean (SD)		18.59 (1.65)		
Attendance to religious activities	739			
Frequently		265 (35.9)	1.29 (0.48–3.42)	1.28 (0.92–1.78)
Never/occasionally		474 (64.1)		
Communication with father	588			
Easily		313 (53.2)	2.29 (0.76–6.85)	1.07 (0.75–1.53)
Not easily		275 (46.8)		
Discuss sexuality with father	593			
Frequently		44 (7.4)	N/A	0.72 (0.37–1.42)
Never/rarely		549 (92.6)		
Communication with mother	726			
Easily		154 (21.2)	1.288 (0.48–3.40)	0.781 (0.52–1.17)
Not easily		572(78.8)		
Discuss sexuality with mother	629			
Frequently		189 (30.00)	4.634 (1.06–20.26)	1.43 (1.01–2.02)
Never/rarely		440 (70.00)		
Have boy/girlfriend	816			
Yes		693 (84.9)	N/A	2.40 (1.57–3.67)
No		123 (15.1)		
Number of sexual partners	686		−0.08 (−0.52 to 0.32)	0.0 (−0.17 to 0.17)
Men: mean (SD)		3.8 (3.08)		
Women: mean (SD)		2.1 (1.61)		
Type of partnership	642			
Important		397 (61.8)	1.45 (0.57–3.68)	2.38 (1.66–3.40)
Not important		245 (38.2)		
Sexual debut	685			
Yes		520 (75.9)	NA	2.63 (1.77–3.91)
No		165 (24.1)		
Age at sexual debut	686		−28 (−0.73 to 0.17)^*^	−0.20 (−0.01 ot 39)^*^
Men: Mean (SD)		16.34 (2.46)		
Women: Mean (SD)		17.23 (1.44)		
Condition of last sexual intercourse	508			
Both willing		474 (93.3)	3.14 (0.86–11.48)	1.25 (0.59–2.63)
Forced		34 (6.7)		
Concerned with STI/HIV acquisition at the last sexual intercourse	516			
Yes		344 (66.3)	1.05 (0.41–2.65)	1.274 (0.85–1.92)
No		175 (33.7)		

^*^Cohen’s d values instead of OR, because age is a continuous variable. Hence, also mean and SD have been reported as descriptive information instead of frequencies

Of the 818 participants that responded to questions about their parents, 50.4% were living in a different household than their father and 41.9% were living in a different household than their mother. Additionally, the father of 170 (20.8%) participants was deceased and of 61 (7.5%) participants, the mother was deceased. Regarding communication, 313 (53.2%) and 154 (21.2%), reported being able to easily communicate with their father and mother, respectively, and 44 (7.4%) and 629 (76.8%) reported discussing sexuality with their father and mother, respectively. Girls were more likely than boys to discuss sexuality with their mother (OR = 2.16, 95% CI 1.54–3.02). Discussing sexuality with one’s mother was associated with VCT service use (OR = 1.43, 95% CI 1.01–2.02).

Of the 816 participants that responded to questions about sexual partnership, 693 (84.9%) reported having a boyfriend or girlfriend, and having a boyfriend or girlfriend was associated with VCT service use (OR = 2.40, 95% CI 1.57–3.67). On average, in their lifetime, male participants had 3.77 (SD = 3.08) sexual partners and women had 2.1 (SD = 1.61). Among those reporting to have a sexual partner, 397 (61.8%) considered their relationship to be important, and this was associated with VCT service use (OR = 2.38, 95% CI 1.66–3.40). A total of 520 participants (75.9%) reported being sexually active, which was associated with VCT service use (OR = 2.63, 95% CI 1.77–3.91). The mean age at sexual debut was 16.34 (SD = 2.46) for men and 17.23 (SD = 1.44) for women. Of the sexually active participants, 344 (66.3%) reported being concerned about getting STI or HIV at their last sexual intercourse, but this was not associated with condom use (OR = 1.05, 95% CI 0.41–2.65) or VCT service use (OR = 1.27, 95% CI 0.85–1.92).

For the 285 (34.80%) participants that had not had a sexual debut, reasons for this were considering sex before marriage sinful (43.5%), being afraid of getting pregnant (39.9%) and being afraid of acquiring an STI or HIV (41.4%). Hence, 43.7% planned to only have sex after getting married and 32.4% reported delaying sex until they meet someone they love.

Regarding preventive sexual behaviours, 199 (96.1%) of male participants and 246 (95.0%) of female participants reported using a condom during their last sex intercourse. VCT services had previously been used by 161 (55.1%) male participants and 226 (57.5%) female participants. When asked when they last used VCT services, 124 (18.1%) indicated having used VCT services in the last 3 months, 81 (11.8%) indicated using VCT in the last 4–6 months, 108 (15.7%) indicated using VCT services a year ago, and 374 (54.4%) did not remember when was the last they visited a health advisor for VCT services.

### Relevant personal determinants for condom use and for VCT service use

**Fig. 1:**
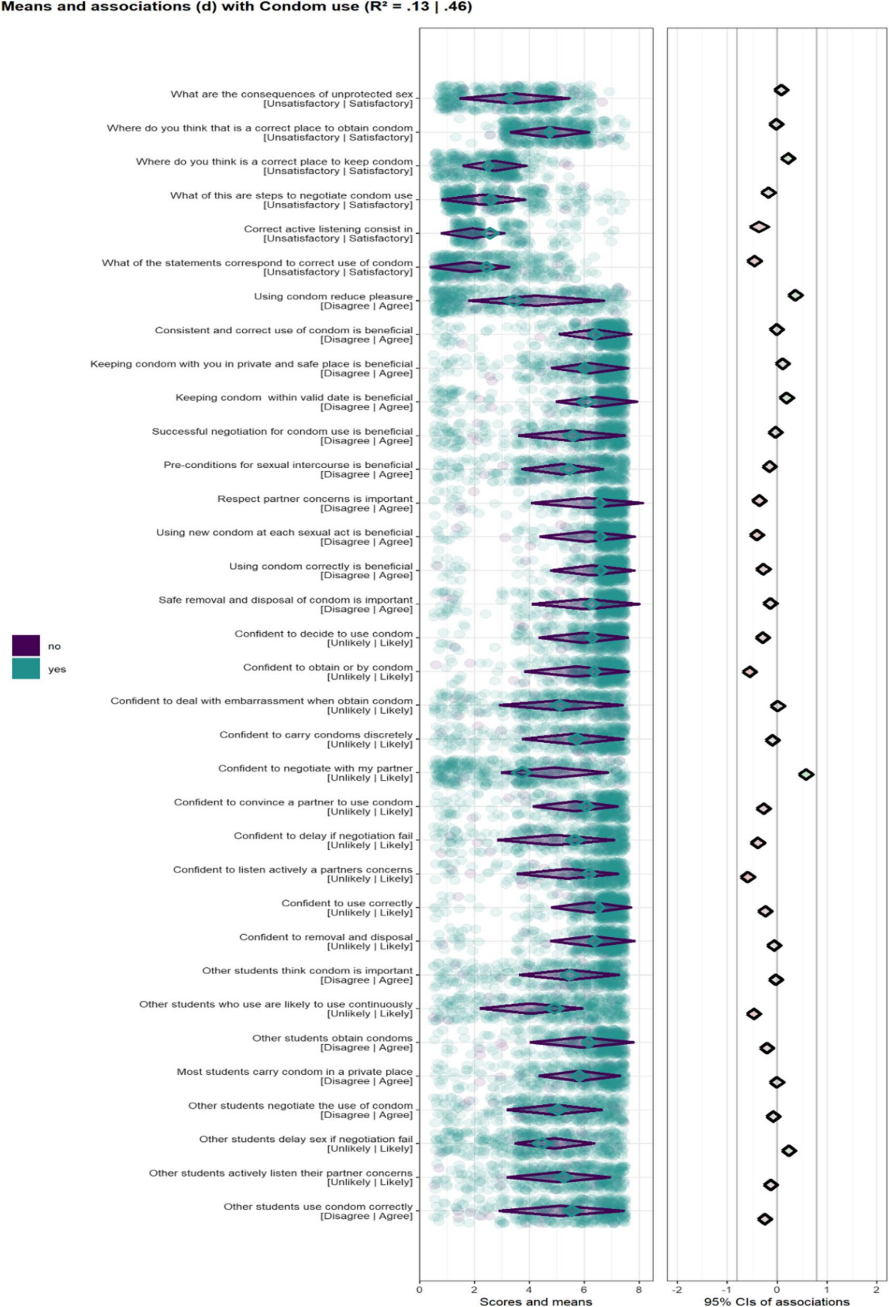
Confidence interval–based estimation of relevance (CIBER) plots for univariate distributions of scores on personal determinants for users (purple) and non-users (green) [left panel] and associations of determinants with condom use [right panel]

**Fig. 2:**
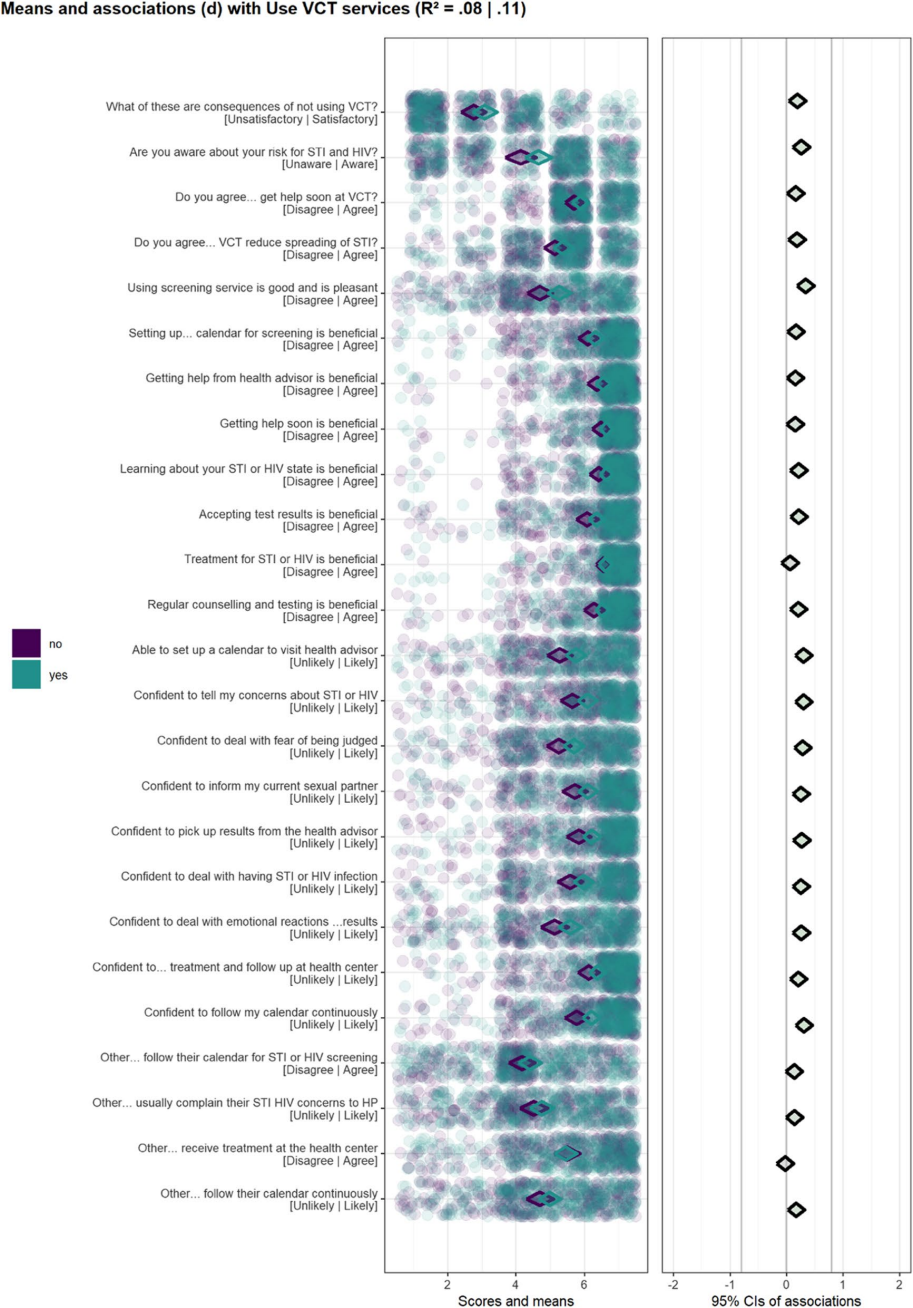
Confidence interval–based estimation of relevance (CIBER) plots for univariate distributions of scores on personal determinants for users (purple) and non-users (green) [left panel] and associations of determinants with use of voluntary counselling and testing services [right panel]

Figures 1 and 2 present the results of CIBER analysis for determinants of condom use and VCT service use, respectively. The items used to assess the score of the behavioural determinants and the anchors are shown on the left of the left-hand panel.

The diamonds in the left-hand panel represent mean scores for items with 99.9% confidence intervals. The stroke colour of the diamond (i.e., the line colour) is indicative of whether participants engage in the behaviour, with turquoise strokes representing participants who had used condom or VCT services and purple strokes representing participants who had not used condoms or VCT services. The position of diamond in the left-hand panel represents the mean score of participants per item with diamonds left having lower mean scores and diamonds to the right having higher mean scores.

The diamonds in the right-hand panel show association strengths (i.e., correlation coefficients) with 95% confidence intervals between individual items and behaviour. The strength and the direction of the association is indicated by the colours of the diamonds, with red diamonds indicating strong and negative associations, green diamonds indicating strong and positive associations, and grey indicating weak associations. The confidence interval of the explained variance (R^2^) of condom use and VCT service use based in all items of determinants are depicted at the top of Figs. 1 and 2, respectively.

### Condom use

We found that participants that used condoms and participants that did not use condoms at last intercourse scored low on knowledge, and moderate to high on subjective norms (Fig. 1). The determinants were negatively and/or weakly associated with condom use (R2 = 0.13–0.46 95% CI) as displayed in the right panel of Fig. 1. Additionally, participants scored moderate to high on attitude and self-efficacy toward condom use, except on one attitude item (Using condoms reduces pleasure) and one self-efficacy item (I am confident to negotiate with my partner). This was more evident in participants who had not used a condom during their last sexual intercourse.

### Voluntary counselling and testing services use

We found that participants scored low to moderate on the majority of items used to measure knowledge and subjective norms, and moderate to high on all items used to measure attitude and self-efficacy toward VCT service use (Fig. 2). In general, all items used to measure the selected personal determinants were not or weakly associated with the VCT use (R^2^ = (0.08–0.11 95% CI). However, one item for knowledge/risk perception (Are you aware of your risk for STI and HIV), one item for attitude (Using screening services is good and pleasant) and three items for self-efficacy (I am able to set up a calendar to visit health advisor; I am confident to deal with fear of being judged; I am confident in ability to deal with an emotional reaction to positive results) were positively associated with VCT service use at 95% CI.

## Discussion

This study set out to assess, in first year university students in Beira city, central Mozambique, the prevalence and determinants of primary and secondary preventive sexual behaviour, namely condom use and voluntary counselling and testing (VCT) services use, using the CIBER analysis approach. Our findings show that almost all sexually active participants used condoms. However, use of voluntary counselling and testing (VCT) services was low. Almost half of male participants and more than a third of female participants had never used VCT services. Of those who had, the majority did not even remember the last time they tested for HIV of STI. This is not surprising given that low use of VCT services has been reported in previous studies (15, 37–39).

In our study, VCT services use was associated with older age, having a boyfriend or a girlfriend, considering one’s sexual relationship to be important, and age at sex debut. This is similar to previous research where VCT service use was found to be related to being sexually active (15, 39), as well as age, gender, religion and age at sex debut (15, 40).

Within our study, the use of VCT services was also associated with discussing sexuality with one’s mother. Unfortunately, in our study, more than half of participants indicated finding it difficult to discuss sexuality with parents. This is concerning as previous studies with AYA in other countries (41–45) have shown that talking about sexuality with parents is related to more preventive sexual behaviour and safer sexual practices.

Unfortunately, in Mozambique and other contexts, talking about sex remains a taboo and is often seen to represent a lack of respect (46–48). Additionally, parents have described it as difficult task (45). Nonetheless, this finding points to the need to improving communication about sexuality between parents and young people.

Widman, Choukas-Bradley (49) describe that parents-adolescents sex communication can be improved through educational efforts combined with practical instructions and technical assistance on language that can be used, and timing. Improvement of parents-adolescents communication can also be achieved when interventions encourage parents to be supportive and communicate openly with their adolescents (50–53). Additionally, research shows that early communication (53) and same-gender communication (54) are desirable for AYA (50).

With regard to personal determinants, our CIBER analyses showed only weak correlations between VCT service use and personal determinants like knowledge, attitude, self-efficacy and subjective norms. The low correlation might be interpreted as result of wrong selection of relevant aspects that can influence VCT services use. To the best of our knowledge as described in methods section, we included personal determinants that we thought might be relevant. However, there is much left for example anticipated regret described as a potential determinant of health behaviour (55).

One of the relevant determinant beyond the level of the adolescents, that should be considered in future studies, is HIV-related stigma, which has previously been described as an important social and/or political determinant of VCT service use (56–61). Unfortunately, HIV-related stigma, measured by means of criminalization of HIV, negative feeling and attitude toward people living with HIV, devaluation, shame/blame/isolation (56) was not directly measured in our survey because, our focus was on personal determinants.

However, some of the beliefs representing the target determinants were associated with VCT service use and should therefore be targeted in future interventions. For example, interventions aiming to increase VCT service use should focus on providing personalized risk information, demonstrating that VCT service use is pleasant and non-judgmental, improving users’ confidence to schedule a visit, and preparing users for possible positive testing results.

In other studies, VCT service use was associated with positive attitude toward people with HIV, higher HIV knowledge, perceived risk of HIV infection, self-efficacy and perception of being health (18, 37, 38, 62).

Our finding that most (almost all) participants had used condoms at last sexual intercourse is not in line with previous studies on condom use in Nigeria, Uganda and Tanzania (14, 30, 63), in which reported condom use was much lower than in our study (ranging from 15% to 59.3%) (13). The high rates of condom use reported by the respondents in our sample may be related to the age of our participants (mean = 19.64 for men; 18.59 for women), and previous exposure to information on the benefits of condom use. Alternatively, it might be due to social desirability bias, with participants over-reporting this normative behaviour (64).

As with VCT services use, condom use was associated with discussing sexuality with one’s mother. The behaviour was also associated with attitude (using condom reduce pleasure) and self-efficacy (confident in ability to negotiate condom use with my partner). These findings are in line with Kalolo and Kibusi (30)’s study on the influence of perceived behaviour control, attitude, and empowerment on reported condom use and intention to use condoms among adolescents in Tanzania. That study reported that condom use was associated with favourable attitudes toward condom use and perceived behavioural control.

Kalolo and Kibusi (30) also found that knowledge and subjective norms were associated with condom use but we did not. This might be related to the type of questions we posed or the way in which our items measuring knowledge and subjective norms were formulated. Possible, the items we drafted inadequately reflected important aspects of knowledge and subjective norms. If so, this could also explain why we found only limited evidence for associations between personal determinants and sexual preventive behaviours.

Although reported condom use was high in our study, we believe that sexual health interventions should still integrate components that reinforce condoms use. In doing so, the focus should be on reinforcing attitudes and self-efficacy. This could be done using modelling, tailoring, reinforcement and technical assistance with educational video tools (65). Additionally, interventions could leverage positive and negative-framed messages on risk communication using brochure visual aids (66).

### Limitations

The findings of this study are subject to some limitations: First, our study design was cross-sectional and thus causal inference about relationships cannot be made. Second, the data in this study are self-reported. Although self-report surveys are widely used to assess sexual behaviour, as results are useful in improving sexual functioning, informing STIs/HIV prevention programmes, they are limited by potential social desirability bias (64, 67, 68). Third, the results of this study may not be generalizable to other AYA as the sample comprised on university students in Mozambique.

Fourth, we acknowledge that CIBER analyses do not consider interactions between variables or examine the impacts of possible confounding variables. We decided to use this approach because it does combine both univariate distributions of determinants and the strength of association with the outcome of interest in a visual and clear fashion, and because it demonstrated its usefulness for the selection of relevant items to improve behavioural outcomes (34, 69, 70). Although it could be argued that interventions ideally target all possible determinants of behaviour, resources are finite and choices must be made. Hence, selecting relevant determinants is required and has an impact on the quantity and quality of intervention content (27, 28).

## Conclusion

New university students are at risk for engaging in sexual risk behaviours. Thus, in first year university students in Beira, Mozambique, reported condom use was high but VCT services were only used by about half of the participants. Interventions aiming to increase VCT service use should focus on improving communication between parents and their adolescent or young adult children, providing personalized risk information, demonstrating that VCT service use is pleasant and non-judgmental, improving users’ confidence to schedule a visit and preparing users for possible positive testing results.

## Data Availability

The datasets generated and/or analysed during the current study are not publicly available because the ethical approval letter does not include public availability of the dataset but, are available from the corresponding author on reasonable request.

## Abbreviations

AYA: Adolescents and young adults
CI: Confidence interval
CIBER: Confidence interval-based estimation of relevance
CIBS: Comité Interinstitucional de Bioética para Saúde
HIV: Human immunodeficiency virus
N/A: Not applicable
OR: Odd ratio
SD: Standard deviation
SPSS: Statistical package for social sciences
STIs: Sexually transmitted infections
VCT: Voluntary counselling and testing
